# Characterization of a novel Jumbo phage JP4 with potential to control pathogenic *Escherichia coli*

**DOI:** 10.1186/s12985-025-03001-4

**Published:** 2025-11-25

**Authors:** Kexin Zhang, Xuemei Wei, He Liu, Bo He, Rui Zhao, Jing Zhou, Jun Deng, Fan Xie, Xu Xiong, Gang Li, Yan Zhao, Jing Wang, Zhenhui Song, Shuguang Lu

**Affiliations:** 1 Immunology Research Center, Medical Research Institute, Southwest University, Chongqing, 400700 China; 2 Department of Microbiology, College of Basic Medical Sciences, Army Medical University, Key Laboratory of Microbial Engineering under the Educational Committee in Chongqing, Chongqing, 400038 China; 3 Department of Cardiology, Xinqiao Hospital, Army Medical University, Chongqing, 400037 China

**Keywords:** Jumbo phage, *Escherichia coli*, Biological identification, Genomic analysis, Antibacterial potential

## Abstract

**Background:**

Amidst rising antimicrobial resistance, bacteriophage (phage) therapy has re-emerged as a pivotal weapon against multidrug-resistant pathogens. Jumbo phages, distinguished by large genomes, show particular therapeutic promise. Yet current understanding of jumbo phages is still lacking.

**Methods:**

Phage was isolated from domestic sewage. The biological properties of JP4 was characterized via transmission electron microscopy, stability tests, one-step growth curve. The genome of JP4 were elucidated by sequencing and bioinformatics tools. Structural proteins were identified via mass spectrometry. Bactericidal and biofilm eradication activities were evaluated using bacterial turbidity measurements and crystal violet assays, respectively. Statistical significance was determined by using one-way ANOVA in GraphPad Prism.

**Results:**

Phage JP4 has an icosahedral head (approximately 110 nm in diameter) and a contractile tail (about 120 nm in length). JP4 possesses a linear dsDNA genome of 370,741 bp, encoding 738 proteins and 8 tRNAs. Phylogenetic analysis revealed that JP4 is a new member of the Asteriusvirus genus, and shares close evolutionary relationships with *Escherichia* phage UB. Additionally, mass spectrometry identified four novel structural protein encoding genes of JP4. Phage JP4 exhibited rapid infection cycle, high stability, potent in vitro bactericidal activity, and strong inhibitory effect on *E. coli* biofilms.

**Conclusions:**

Phage JP4 is a new member of the Asteriusvirus genus. As a lytic jumbo phage with rapid bactericidal activity and strong biofilm degradation capacity, JP4 is a promising therapeutic candidate against *E. coli* O157:H7 infections. This study provides insights into the diversity and clinical potential of jumbo phages in combating pathogens.

**Supplementary Information:**

The online version contains supplementary material available at 10.1186/s12985-025-03001-4.

## Background

Foodborne bacterial pathogens continue to pose a formidable challenge to global health security. Epidemiological surveillance data from the World Health Organization (WHO) reveal an annual burden of 600 million foodborne illness cases worldwide, culminating in approximately 420,000 deaths [1]. While *Escherichia coli* (*E. coli*), *Listeria monocytogenes*, and *Salmonella* contribute significantly to this crisis. As a natural commensal in mammalian gut microbiota [2], *E. coli* can acquire virulence genes through horizontal gene transfer (HGT), and transform into pathogenic strains such as enteropathogenic *E. coli* (EPEC), enterotoxigenic *E. coli* (ETEC), and enterohemorrhagic *E. coli* (EHEC) [3–6]. Since the early 1980 s, pathogenic *E. coli* has emerged as a predominant foodborne pathogen [7], which can induce various diseases including enteritis, food poisoning, and hemolytic uremic syndrome [8]. Current therapeutic regimens employing β-lactams (ampicillin, cefotaxime) and fluoroquinolones (ciprofloxacin) [7, 9, 10] face dual challenges: inherent resistance mechanisms encoded on mobile genetic elements [11], and the paradoxical risk of toxin release during bacteriolysis - a phenomenon exacerbating clinical manifestations of hemolytic uremic syndrome and hemorrhagic colitis. This therapeutic dilemma underscores the critical need for novel and precision antimicrobial strategies that circumvent traditional microbicidal mechanisms.

Bacteriophages (phages), as obligate bacterial viruses, demonstrate exquisite host tropism through receptor-mediated infection mechanisms, thus preserving commensal microbiota integrity during therapeutic applications [12]. Phages dominate the virosphere, with metagenomic census data revealing their ubiquitous distribution across terrestrial/aquatic biomes and mammalian microbiomes [13]. While most tailed phages (Caudoviricetes) maintain compact genomes typically spanning 2 kb-200 kb, phageomics has uncovered a distinct cohort of jumbo phages (≥ 200 kb genomes) inhabiting nutrient-dense niches from deep-sea vents to human colonic crypts [14, 15]. The prototypical jumbo phage phiKZ was discovered in 1978, with a genome size of 280 kb [16, 17]. Recent discoveries have expanded this frontier, with the discovery of the largest known phage genome size of 735 kb [18]. Conventional phage isolation methods systematically undersample these jumbo phages due to their low diffusion coefficients in semisolid media and retention by bacterial-grade filters [19–21]. With the advancements of phage isolation technology, an increasing number of jumbo phages have been successfully isolated, most of which target Gram-negative bacteria [20, 22, 23].

The genomes of jumbo phages encode sophisticated genetic arsenals that confer metabolic autonomy and evolutionary plasticity. Comparative genomic analyses reveal expanded orthologous groups in DNA replication machinery, nucleotide metabolism pathways, transcription regulation, and structural proteomes, enabling temporal control of life cycles independent of host transcriptional machinery [19, 24]. Crucially, these phages deploy innovative anti-defense strategies including self-assembled phage nucleus structures that compartmentalize phage replication form bacterial immunity systems. These evolutionary adaptations position jumbo phages as next-generation antimicrobial platforms capable of overcoming pan-drug-resistant pathogens [25]. Nevertheless, research on jumbo phages still faces several challenges, primarily due to the relatively recent onset of investigations, the limited number of isolated jumbo phages, and incomplete jumbo phage genome annotations, which restrict our understanding of jumbo phages and impede the full realization of their application potential. Consequently, the isolation and characterization of novel jumbo phages remain a critical foundation for advancing this field and hold significant implications for the future development of jumbo phages.

In this study, a lytic jumbo phage was successfully isolated from domestic sewage using *E. coli* O157:H7 as the host bacterium. The biological properties, genomic features, structural proteins, and phylogenetic relationships of jumbo phage JP4 were characterized. In vitro experiments demonstrated that phage JP4 exhibited potent lytic activity against *E. coli* and effectively disrupted biofilm formation, indicating that JP4 holds promise as a potential antibacterial agent for combating infections caused by pathogenic *E. coli* strains.

## Materials and methods

### Bacterial strains and culture conditions

The detailed information regarding the bacterial strains utilized in this study is provided in Table S1. These bacterial strains were retrieved from liquid nitrogen storage and streaked onto solid LB agar plates using the three-line method. The plates were incubated at 37 °C overnight to allow single colonies to form. A single colony was selected using a sterile inoculation loop and transferred into 3 mL of liquid LB medium. The cultures were grown with shaking at 37 °C and 220 rpm for 2.5 to 4 h until they reached the logarithmic growth phase (OD_600_ = 0.5, corresponding to approximately 1 × 10^8^ CFU/mL). The cultures were then stored at 4 °C for subsequent experiments. For further use, 10 µL of the bacterial suspension was inoculated into 3 mL of fresh liquid LB medium and shaken at 37 °C and 220 rpm for 2 to 3.5 h until the OD_600_ reached approximately 0.3 (for phage inoculation) or 0.5 (for double-layer agar culture). All procedures were performed under aseptic conditions in a laminar flow hood.

### Phage isolation and efficiency of plating (EOP) determination

Phage particles were isolated and purified using an optimized protocol adapted from established methodologies [26]. Domestic wastewater (200 mL; Shapingba District, Chongqing, China) was subjected to centrifugation at 5000 × g for 10 min to pellet particulate matter. The clarified supernatant was sterile-filtered through a 0.45 μm membrane (Sangon, Shanghai) to eliminate bacterial contaminants. The filtrate was combined with 200 mL of log-phase host bacterial culture (OD_600_ ≈ 0.25) and incubated overnight at 37 ℃ with shaking (200 rpm). For phage enrichment, 3 mL of the co-culture was centrifuged (5,000 × g, 10 min), and the supernatant was filtered (0.45 μm) to obtain a phage-enriched lysate. Plaque formation and efficiency of plating (EOP) were assessed via the double-layer agar plaque assay: 50 µL of lysate was mixed with 200 µL of fresh host culture (OD_600_ ≈ 0.125), incorporated into 3.5 mL of molten top agar (0.3% agar, 45 °C), and overlaid onto LB agar plates. Following solidification, plates were incubated at 37 °C for 6–8 h to visualize discrete plaques. Distinct plaques were excised and suspended in 3 mL of log-phase host culture for secondary amplification (37 °C, 5 h). Cellular debris was removed by centrifugation (10,000 × g, 10 min), and the clarified lysate was serially diluted in LB broth. Plaque-forming units (PFU) were quantified using the double-layer assay, with plaque counts performed at a gradient dilutions.

### Purification of phage particles

The phage particles were purified by the method reported previously [27]. In brief, the phage supernatant (10^10^ CFU/mL) was mixed with host bacteria in logarithmic-phase and cultured until complete lysis. Subsequently, DNase Ⅰ (5 µg/mL) and RNase A (10 µg/mL) were added to achieve final concentrations of 1 µg/mL each to inactivate the host bacterial nucleic acids, followed by the addition of NaCl (5.84 g/100 mL) to precipitate bacterial debris. The mixture was then centrifuged at 10,000 g for 10 min, and the supernatant was collected. To concentrate the phages, solid PEG 8000 (polyethylene glycol 8000) was added to the upper clear layer to a final concentration of 10% (w/v), ice bath for over an hour, followed by centrifugation at 12,000 g for 10 min. The precipitate was resuspended in in 2 mL of TM (tris-magnesium sulfate) buffer (pH 7.5). Subsequently, the mixture was added with chloroform and centrifuged at 5,000 g for 10 min. The upper aqueous phase was collected, and this extraction step was repeated 3 times to obtain crude phage particles. A cesium chloride gradient was established by sequentially adding solutions of different densities to centrifuge tubes. The crude phage suspension was carefully added on the top of the gradient. Ultracentrifugation was performed at 100,000 × g for 20 h at 4 ℃. Owing to the differences in buoyant density, the phages migrated to a distinct white band within the density range of 1.4–1.6 g/mL. This band was collected using a fine needle tip, then cesium chloride was removed by dialysis.

### Transmission electron microscopy (TEM) observation

Phage JP4 morphology was analyzed through transmission electron microscopy performed by Lilai Biotechnology (Chengdu, China). Samples were prepared using a standardized negative-staining protocol: Purified phage suspensions were adsorbed onto carbon-coated copper grids for 10 min at room temperature. Excess liquid was removed by capillary action using Whatman filter paper (Solarbio, Beijing), followed by negative staining with 1% (w/v) aqueous phosphotungstic acid (PTA, pH 6.8) for 20 s. Grids were air-dried overnight in a desiccator before imaging. Microstructural analysis was conducted using a transmission electron microscope (JEM-1400FLASH, Japan) operated at 80 kV accelerating voltage. Three independent grid preparations were examined to confirm morphological consistency.

### Determination of the multiplicity of infection (MOI) and one-step growth curve

To establish the optimal MOI, phage-host interactions were evaluated across six logarithmic dilutions (MOI: 10² to 10⁻³). Phage suspensions were mixed with mid-log phase host cultures (OD_600_ = 0.4 ± 0.05) in 1:10 (v/v) ratio and incubated at 37 °C with shaking (200 rpm) for 5 h. Following chloroform treatment (1% v/v) to ensure complete cell lysis, lysates were subjected to tenfold serial dilutions in PBS (pH 7.4) containing 10 mM MgSO4. Triplicate plaque assays were performed using the double-layer agar method (0.3% top agar), with efficiency of plating (EOP) quantified after 6 h incubation at 37 °C. The MOI yielding maximal plaque-forming units (PFU) was selected for subsequent growth curve analysis. One-step growth kinetics were characterized using an adsorption-inhibition protocol [28]. Briefly, phage-host mixtures (MOI optimized) underwent 5 min adsorption at 4 °C to synchronize infection, followed by centrifugation (10,000 × g, 4 °C, 10 min) to remove unadsorbed virions. The pellet was resuspended in prewarmed LB supplemented with 10 mM CaCl2. Aliquots collected at 10 min intervals (0–120 min) were immediately treated with 0.22 μm-filtered phage inactivation buffer (10 mM EDTA, 0.1% sodium deoxycholate) to halt replication. Processed samples were diluted in PBS-Tween 20 (0.02%) and plated using the standard overlay method. These experiments were repeated three times.

### Stability testing of phages

Phage stability was assessed through four environmental stress challenges. pH tolerance was evaluated using PBS buffer adjusted to pH values ranging from 1 to 12 [29]. Phage supernatants (~ 10^10^ PFU/mL final concentration) were incubated 1 h in pre-equilibrated buffers with gentle rotation (30 rpm). Surviving phage particles were immediately neutralized in Tris-HCl (pH 7.5) prior to dilution and plating. For chloroform resistance, phage suspensions (10^10^ PFU/mL) were treated with chloroform (1:1 v/v) for 2 min with vortex mixing (5 s pulses, 3 ×). Treated lysates underwent tenfold serial dilutions in PBS before EOP quantification via standard overlay assay. Thermal stability testing employed calibrated water baths (± 0.5 °C) across seven temperatures (4 °C, 37 °C, 50 °C, 60 °C, 70 °C, 80 °C, and 90 °C) [30]. Phage samples (~ 10^10^ CFU/mL) were heat-shocked for 1 h. Residual infectivity was assessed after rapid cooling (4 °C, 5 min). UV resistance was measured using a 254 nm germicidal lamp (15 W/m²). Phage lysates in open Petri dishes (20 cm irradiation distance) were sampled at 10 min intervals (0–60 min). Dark control samples maintained parallel processing to exclude light-independent inactivation.

### Detection of the host range of phage JP4

Tested bacterial strains (Table S1) were grown in LB broth at 37 °C with shaking (200 rpm) to mid-log phase (OD_600_ = 0.4 ± 0.1). Phage suspensions were combined 1:1 (v/v) with bacterial cultures in sterile 1.5 mL microcentrifuge tubes and incubated at room temperature for 15 min to facilitate adsorption. Tenfold serial dilutions were prepared, and 10 µL aliquots from each dilution were spot-plated onto pre-dried LB agar plates. Plates were incubated statically at 37 °C and assessed for confluent lysis zones. Lytic activity was scored qualitatively: “+” indicated complete clearing, “-” denoted no observable effect. Biological triplicates were performed using independent bacterial cultures and phage preparations.

### Extraction and purification of phage genomic nucleic acid

Genomic DNA extraction from phage JP4 employed a modified phenol-chloroform method [31] optimized for jumbo phage virions. High-titer phage lysates (~ 1.2 × 10^11^ PFU/mL) underwent sequential enzymatic treatment: DNase I (10 U/mL) and RNase A (50 µg/mL) in 10 mM MgCl₂ (37 °C, 30 min) to degrade free nucleic acids, followed by EDTA (0.5 mmol/mL) inactivation (65 °C, 30 min). Viral capsids were lysed using proteinase K (1 mg/mL in 0.5% SDS) at 56 °C for 60 min. Nucleic acid extraction involved three PCI (phenol: chloroform: isoamyl alcohol 25:24:1) cycles. Each cycle consisted of vigorous vortexing (30 s), centrifugation (5,000 × g, 10 min, 4 °C), and aqueous phase recovery. Residual phenol was removed through chloroform back-extraction (1:1 v/v). DNA precipitation utilized 0.1 volumes 3 M sodium acetate (pH 5.2) and 2.5 volumes ice-cold ethanol (−20 °C, 1 h). The DNA pellet obtained by centrifugation (12,000 × g, 10 min, 4 °C) underwent two 70% ethanol washes, air-dried in a UV-sterilized laminar flow hood (15 min), and resuspended in nuclease-free TE buffer (10 mM Tris-HCl, 1 mM EDTA, pH 8.0). DNA integrity was confirmed via 0.8% agarose gel electrophoresis (5 V/cm, 45 min) with λ-HindIII markers. Purified DNA was stored at −20 °C in amber vials with desiccant.

### Genome sequencing and annotation

The genomic DNA of phage JP4 was subjected to whole-genome sequencing by Personal Biotechnology Co., Ltd. (Shanghai, China). The whole-genome shotgun (WGS) approach was used to construct libraries with varying insert sizes. These libraries were sequenced using the Illumina NovaSeq platform in paired-end (PE) mode. Read data were processed for quality control and adapter trimming with fastp (https://github.com/OpenGene/fastp). The whole genome was assembled using SPAdes v. 3.5.0 software [32]. The terminal types of the phage genomic DNA were predicted using PhageTerm software [33]. The JP4 genome sequence was annotated using the RAST Server (https://rast.nmpdr.org/rast.cgi) [34], and the annotation results were validated using GeneMark (https://exon.gatech.edu/) [35] and BLAST [36]. The tRNA-coding genes in the JP4 genome were predicted using tRNAscan-SE 2.0 (http://trna.ucsc.edu/tRNAscan-SE/) [37, 38]. The annotation data of JP4 were submitted to GenBank (https://www.ncbi.nlm.nih.gov/genbank/) [39] via BankIt (https://www.ncbi.nlm.nih.gov/WebSub/). The genome map of JP4 was visualized using Proksee (https://proksee.ca/) [40] and BRIG software [41]. Potential virulence factors in the JP4 genome were predicted using VFDB (https://www.mgc.ac.cn/cgi-bin/VFs/v5/main.cgi) [42], while drug resistance genes were identified using ResFinder 4.6.0 (http://genepi.food.dtu.dk/resfinder) [43]. The life cycle of phage JP4 was predicted using DeePhage (http://cqb2.pku.edu.cn/ZhuLab/DeePhage/) [44].

### Comparative genomics analysis

Comparative genomics analysis was initiated by performing BLAST alignments [36] of phage JP4 whole genome sequence against the NCBI viral RefSeq database (March 11, 2025) and PhageScope database (https://phagescope.deepomics.org/) [45]. Twenty phages exhibiting reciprocal best hits were selected for synteny analysis. Whole-genome comparisons were executed using tBlastX with subsequent visualization through EasyFig (http://mjsull.github.io/Easyfig/) [46]. BRIG (BLAST Ring Image Generator, https://sourceforge.net/projects/brig/) comparison of complete genome sequences of JP4 with other 20 related phages was performed. For evolutionary contextualization, the VICTOR online tool (https://ggdc.dsmz.de/victor.php) [47] was used to perform phylogenetic analysis of 52 phage sequences with similarity to phage JP4. Taxonomic classification of phage JP4 and the calculation of average nucleotide identity (ANI) were performed using taxMyPhage (https://ptax.ku.dk/) [48].

### Identification of structural proteins of phage JP4

The structural proteins of phage JP4 were analyzed following established protocols [27]. Purified phage particles were first suspended in Laemmli buffer and denatured by boiling at 100 °C for 5 min, followed by immediate cooling on ice. Proteins were separated through sodium dodecyl sulfate-polyacrylamide gel electrophoresis (SDS-PAGE) using 12% polyacrylamide gels under standard electrophoretic conditions. After Coomassie blue staining, twenty distinct protein bands (~ 3 mm width each) were carefully excised from the gel. These gel slices underwent sequential processing: destaining with 50% acetonitrile/50 mM ammonium bicarbonate solution, reduction with dithiothreitol (10 mM, 30 min at 56 °C), alkylation with iodoacetamide (55 mM, 20 min in dark), and overnight trypsin digestion (12.5 ng/µL in 50 mM ammonium bicarbonate). Purified phage JP4 particles were resuspended in SDS solution (final concentration 10% w/v) and heated at 100 °C for 10 min to achieve complete denaturation, followed by overnight digestion with trypsin (12.5 ng/µL in 50 mM ammonium bicarbonate). The above resulting peptides were desalted and analyzed using a time-of-flight mass spectrometer (Q Exactive™ HF-X, Thermo Scientific). Acquired spectra were processed through Proteome Discoverer 2.4 software, searching against the JP4 genome-derived protein database with tryptic digestion parameters. Identified proteins were mapped to their corresponding gel bands through molecular weight alignment, with false discovery rates maintained below 1% using reverse decoy databases.

### In vitro bactericidal assay

Mid-log phase *E. coli* O157:H7 cultures (OD_600_ 0.30 ± 0.02, 1.2 × 10^6^ CFU/mL in LB broth) were aliquoted (180 µL/well) into sterile 96-well plates. Phage JP4 suspensions underwent tenfold serially dilutions (10^9^−10^1^ PFU/mL) in PBS (pH 7.4), with 20 µL added per test well. Blank wells contained 20 µL PBS (pH 7.4). The plate was incubated statically at 37 °C in a microplate reader, with OD_600_ measurements recorded hourly for 12 h. Triplicate measurements were performed for all test conditions.

### Biofilm eradication test

To evaluate biofilm inhibition, host bacteria were grown to mid-log phase (OD_600_ = 1.4–1.6, ~ 5 h). Cells were harvested by centrifugation (5,000 × g, 10 min) and resuspended in fresh LB broth containing 1% glucose. The suspension was adjusted to 10^8^ CFU/mL and dispensed into a 96-well plate (200 µL/well), with LB-only wells serving as blanks. After 24 h static incubation at 37 °C, spent media were carefully aspirated. Following a preliminary pre-experiment, it was determined that the minimum inhibitory concentration (MIC) of ampicillin is 100 mg/mL. Test wells received 200 µL of either serially diluted phage lysates or ampicillin (concentration gradient spanning 0.25 × to 4 × MIC), while control wells retained untreated bacterial suspension. Following 12 h treatment, wells were gently washed twice with PBS to remove non-adherent cells. Biofilm quantification employed crystal violet staining: wells were stained with 0.1% crystal violet for 15 min, rinsed with distilled water, air-dried, and destained with 95% ethanol for 30 min. Absorbance at 570 nm was measured, with biofilm formation percentage calculated as [(OD_570_ of bacterial suspension - OD_570_ of blank)/(OD_570_ of untreated samples- OD_570_ of blank)] × 100%. All conditions included triplicate biological replicates.

### Statistical analysis methods

Statistical analyses were performed using one-way ANOVA in GraphPad Prism 9.5.1. Significance thresholds were set at *P* < 0.05 (significant) and *P* < 0.01 (highly significant), with Tukey’s post hoc test applied for multi-group comparisons. Data represent means ± SD from three independent experiments.

## Results

### The biological characteristics of phage JP4

**Fig. 1:**
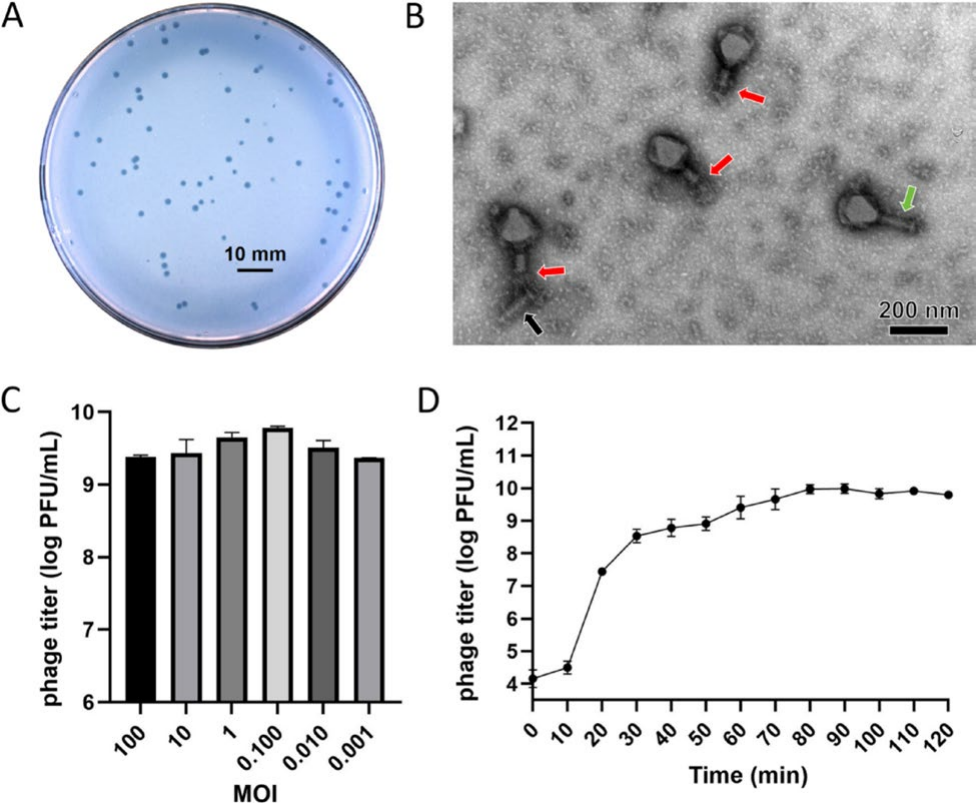
Characteristics of phage JP4. **A** Plaques formed by JP4. Scale bar: 10 mm. **B** Transmission electron microscopy (TEM) images of JP4 particles. The green arrow indicates an intact tail, while the red arrows highlight contracted tails. The black arrow points to a tail detached from the head. Magnification: 100,000×, accelerating voltage: 80 kV. Scale bar: 200 nm. **C** Multiplicity of infection (MOI) determination for phage JP4. **D** One-step growth curve of JP4. All experiments were conducted in triplicate

Phage JP4 was isolated from wastewater using *E. coli* O157:H7 as the primary host. It produced small, transparent plaques (1.5 ± 0.2 mm diameter) on host lawns (Fig. 1A). Among 68 clinical *E. coli* isolates tested, three strains showed susceptibility, while no lytic activity was observed against *Shigella dysenteriae*, *Salmonella typhi*, or *Staphylococcus aureus* (Table S1). Transmission electron microscopy revealed JP4’s characteristic myovirus morphology, featuring an icosahedral capsid (110 ± 5 nm diameter) and a contractile tail (120 ± 8 nm length) (Fig. 1B). Replication efficiency varied with multiplicity of infection (MOI), peaking at MOI = 0.1 (Fig. 1C). One-step growth analysis indicated a 10-minute latent phase followed by a 30-minute burst period (Fig. 1D). The burst size, defined as the average number of phage particles released per infected cell, was estimated to be 57 for phage JP4.

### Stability of phage JP4

**Fig. 2:**
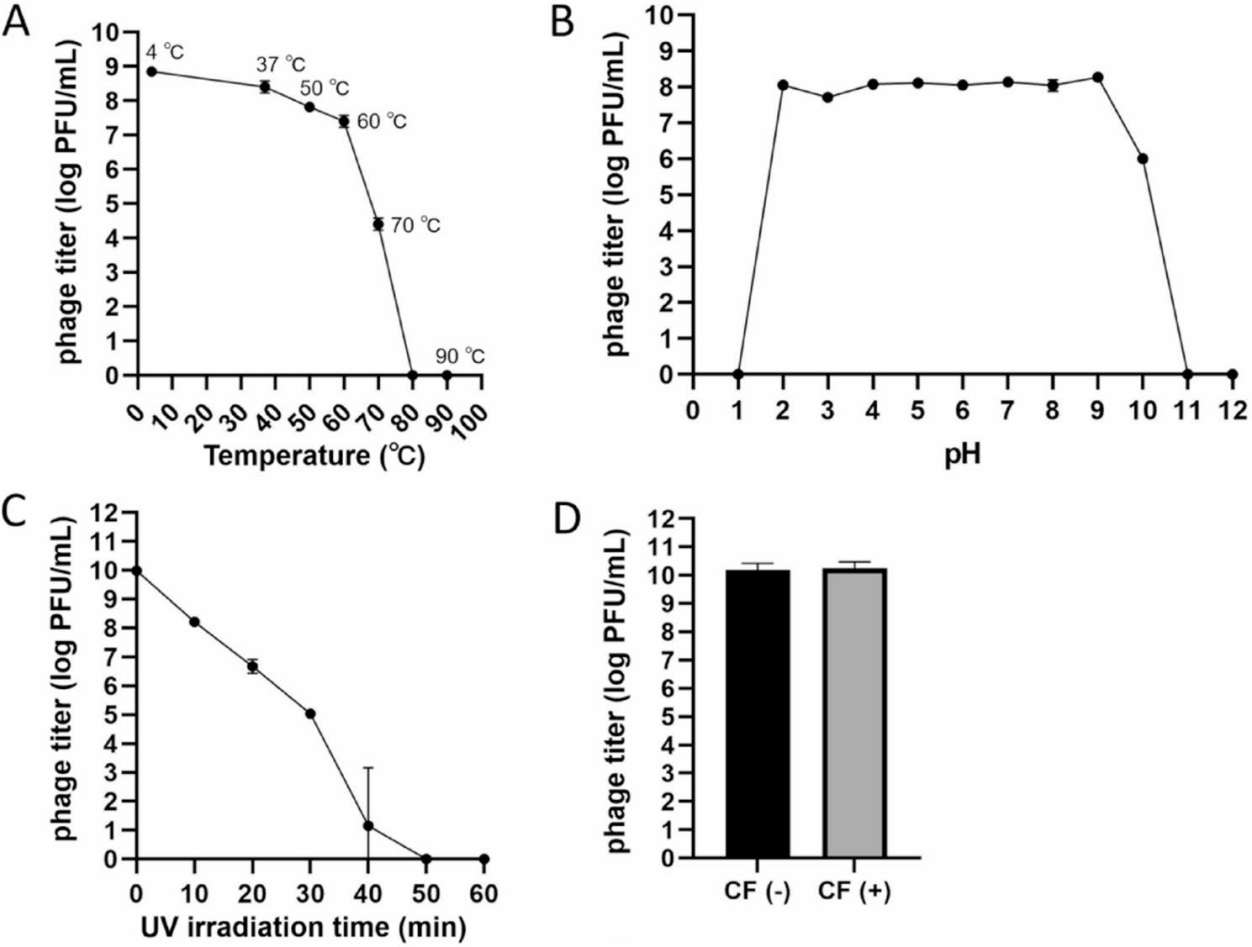
Physical and chemical stability of phage JP4. **A** Temperature stability of JP4. **B** pH stability of JP4. **C** Ultraviolet (UV) stability of JP4. **D** Chloroform sensitivity of JP4. CF: chloroform. All experiments were conducted in triplicate to evaluate the stability and sensitivity under different conditions

JP4 demonstrated thermal resilience up to 60 °C (incubation for 60 min), retaining ~ 82.3 ± 5.0% viability (0.5 × 10^8^ PFU/mL baseline). Virion integrity collapsed at > 60 °C, with complete inactivation at 80 °C (Fig. 2A). Broad pH tolerance (2.0–10.0) maintained ≥ 1.2 × 10^6^ PFU/mL of JP4 (Fig. 2B), though extremes (pH < 2/pH > 10) caused capsid denaturation. Ultraviolet exposure caused progressive inactivation of JP4, with complete loss of infectivity following 50-minute irradiation (Fig. 2C). Chloroform treatment showed no measurable impact on phage viability (Fig. 2D).

### Genome of phage JP4

**Fig. 3:**
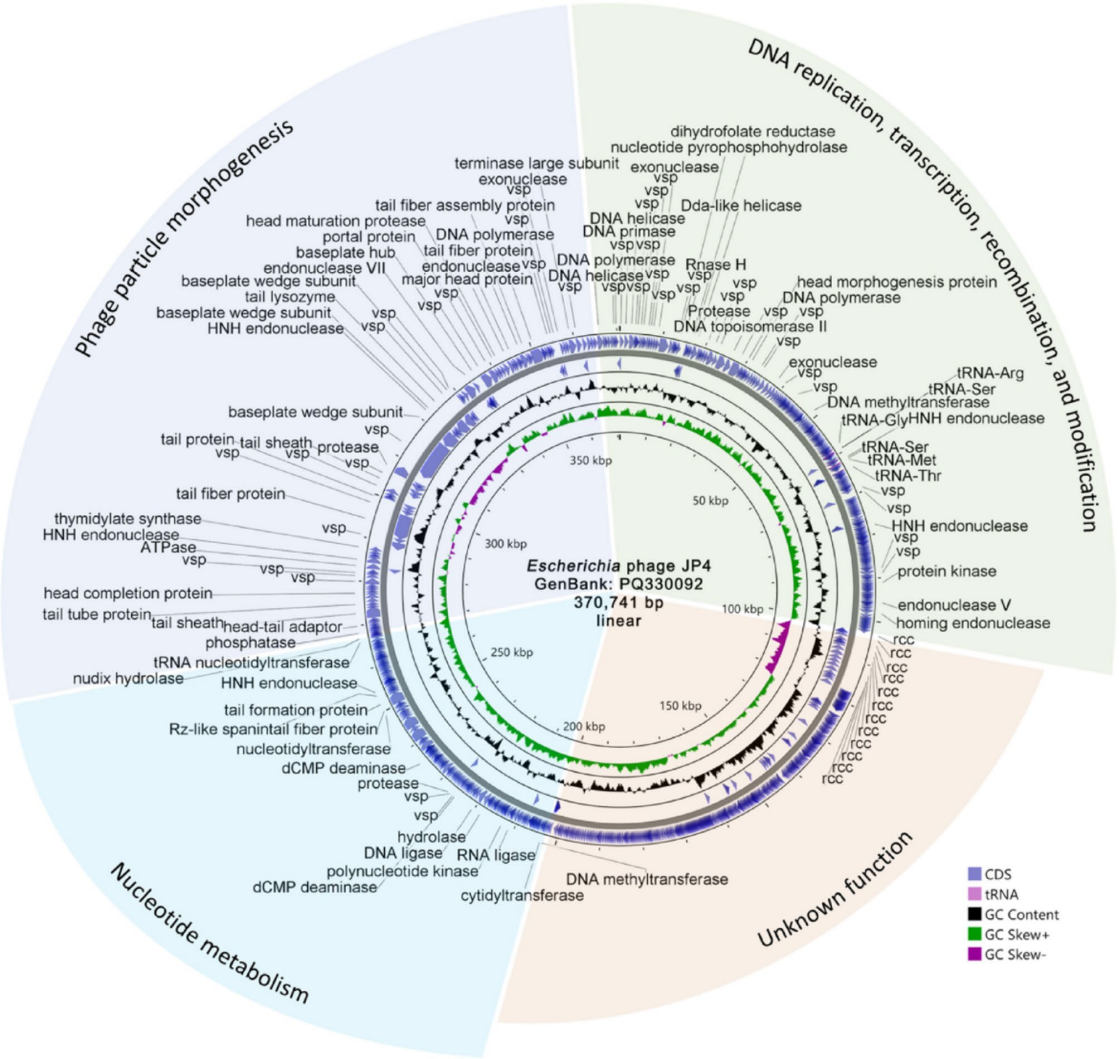
Genomic map of phage JP4. The outermost circle represents genes encoded by the positive strand, followed by those encoded by the negative strand. Different color blocks represent different functional modules. rcc, regulator of chromosome condensation; vsp, virion structural protein

Whole-genome sequencing analysis revealed that the genome of phage JP4 is 370,741 bp in length (GenBank accession number: PQ330092) (Fig. 3), classifying it as a jumbo phage. The sequencing depth reached 3,588-fold, ensuring uniform coverage across the entire genome of JP4. Prediction using PhageTerm software [33] indicated that the genome DNA of JP4 is a linear molecule with cos (cohesive) ends. The JP4 genome has a G + C content of 34.32%, and encodes 738 proteins and 8 tRNAs. Among these, 161 proteins were predicted to have functions, accounting for 21.8% of all proteins. Based on the known functions of proteins and in accordance with the modular structural characteristics of the phage genomes, the JP4 genome was divided into four functional modules (Fig. 3), among which the function of one module remains unclear. Based on the prediction results from DeePhage [44], phage JP4 is a lytic phage and does not encode integrases, excisionases, transposases, or other related functions. No genes associated with drug resistance or virulence factors were identified in the genome of phage JP4.

### Identification of structural proteins of phage JP4

**Fig. 4:**
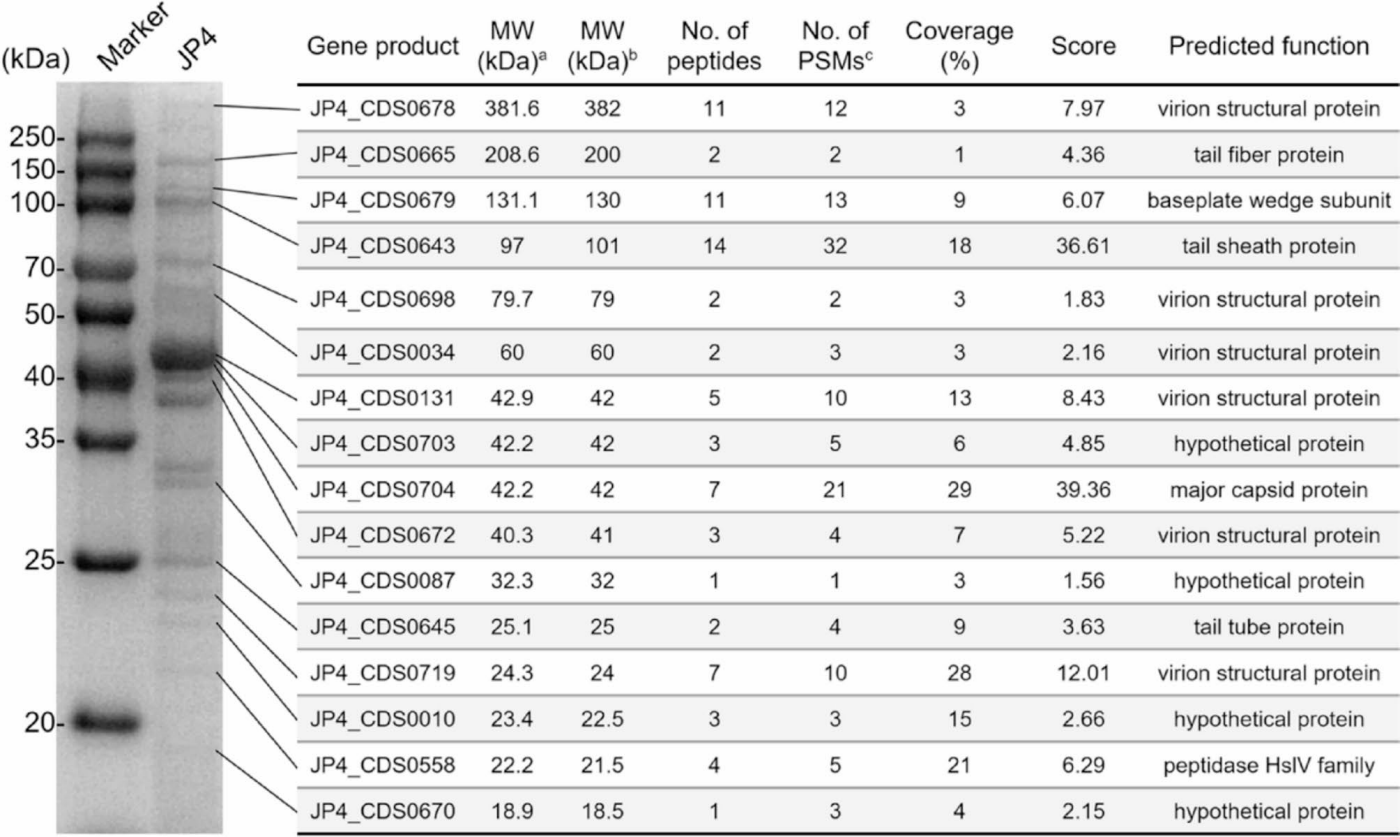
SDS-PAGE and mass spectrometry identification of JP4 structural proteins. MW: molecular weight. ^a^MW value was theoretically calculated. ^b^MW value was experimentally estimated. ^c^Peptide segments matched to the secondary spectrum

LC-MS/MS analysis of structural proteins detected 49 proteins encoded by JP4 (Supplementary datasets 1). SDS-PAGE analysis of JP4 virions resolved 20 protein bands (18–385 kDa) (Fig. S1 and Fig. 4), with the dominant 42 kDa band containing the major capsid protein. Eighteen protein bands were matched to predicted ORFs via Mascot searches. Four previously annotated hypothetical proteins (JP4_CDS0703, JP4_CDS0087, JP4_CDS0010, and JP4_CDS0670) were validated as structural components through peptide-spectrum matches (Fig. 4).

### Comparative genomic analysis of phage JP4

**Fig. 5:**
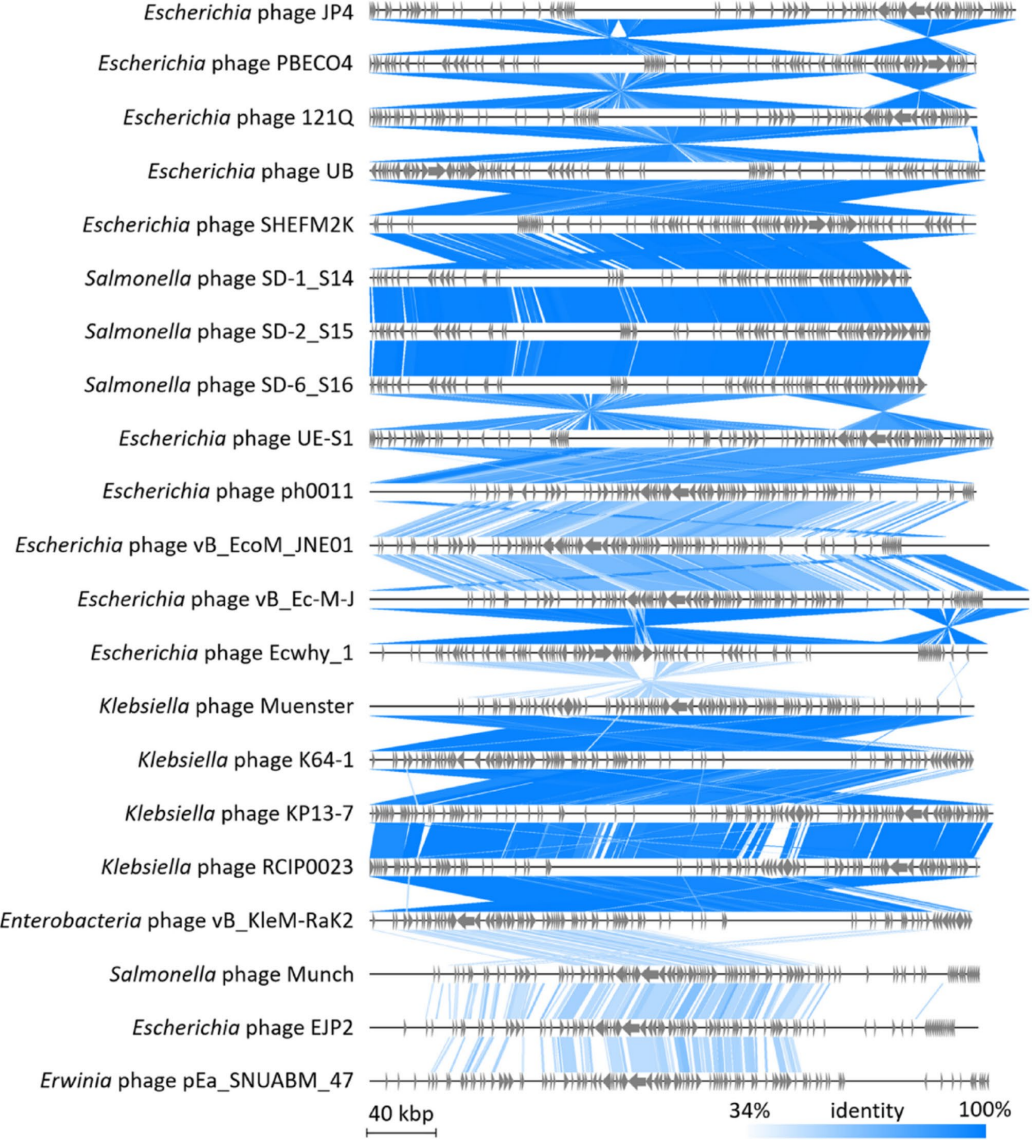
tBlastX alignment of phage JP4. The identity cut-off is set at 34%. The funnel-shaped structure represents the reverse complementary configuration of the nucleotide sequence

**Fig. 6:**
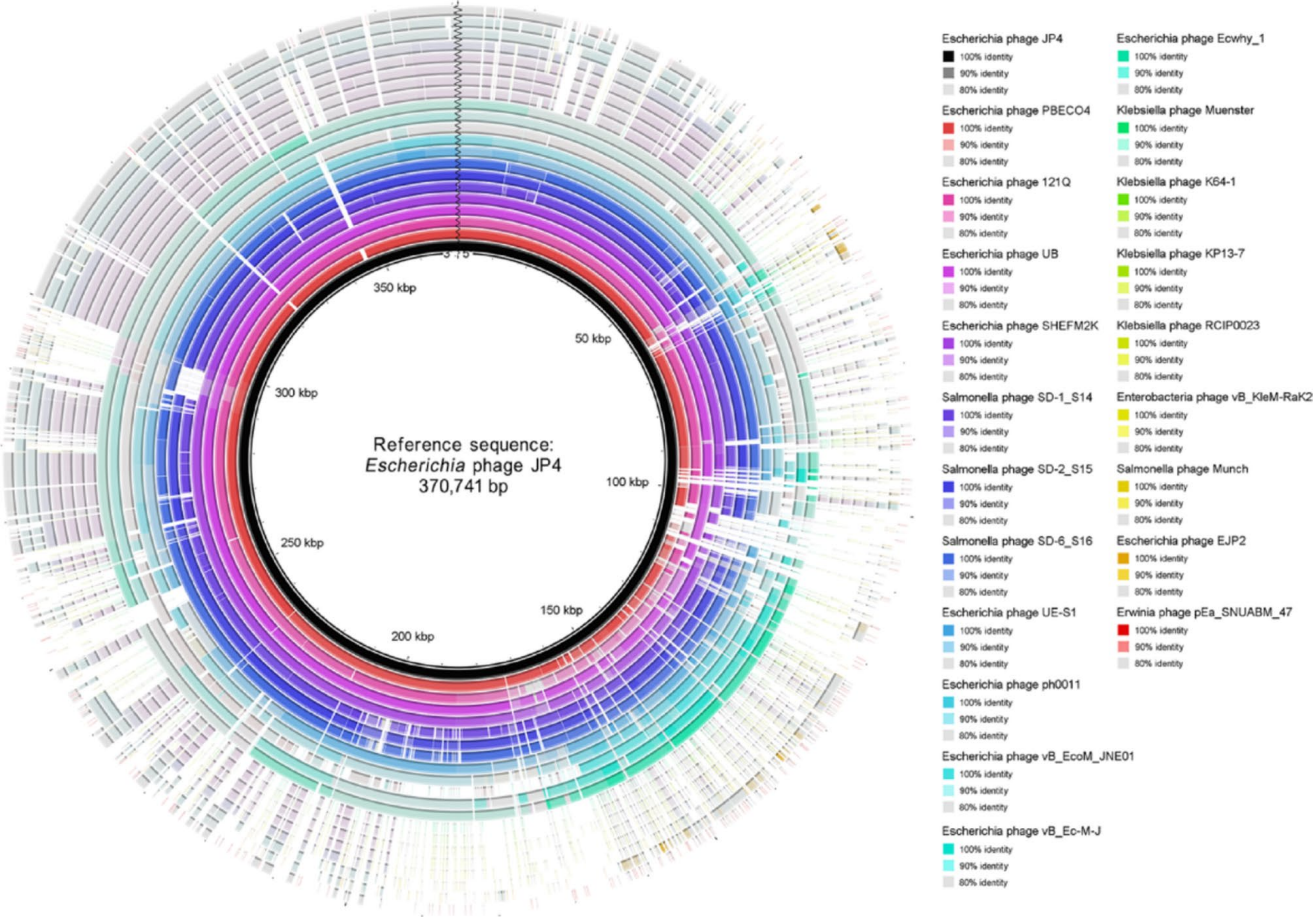
BRIG comparison of complete genome sequences of JP4 with other 20 related phages showed in Fig. 5. Identity labels of the 21 phage genomes are shown in the same order as the rings from the innermost to the outermost

**Fig. 7:**
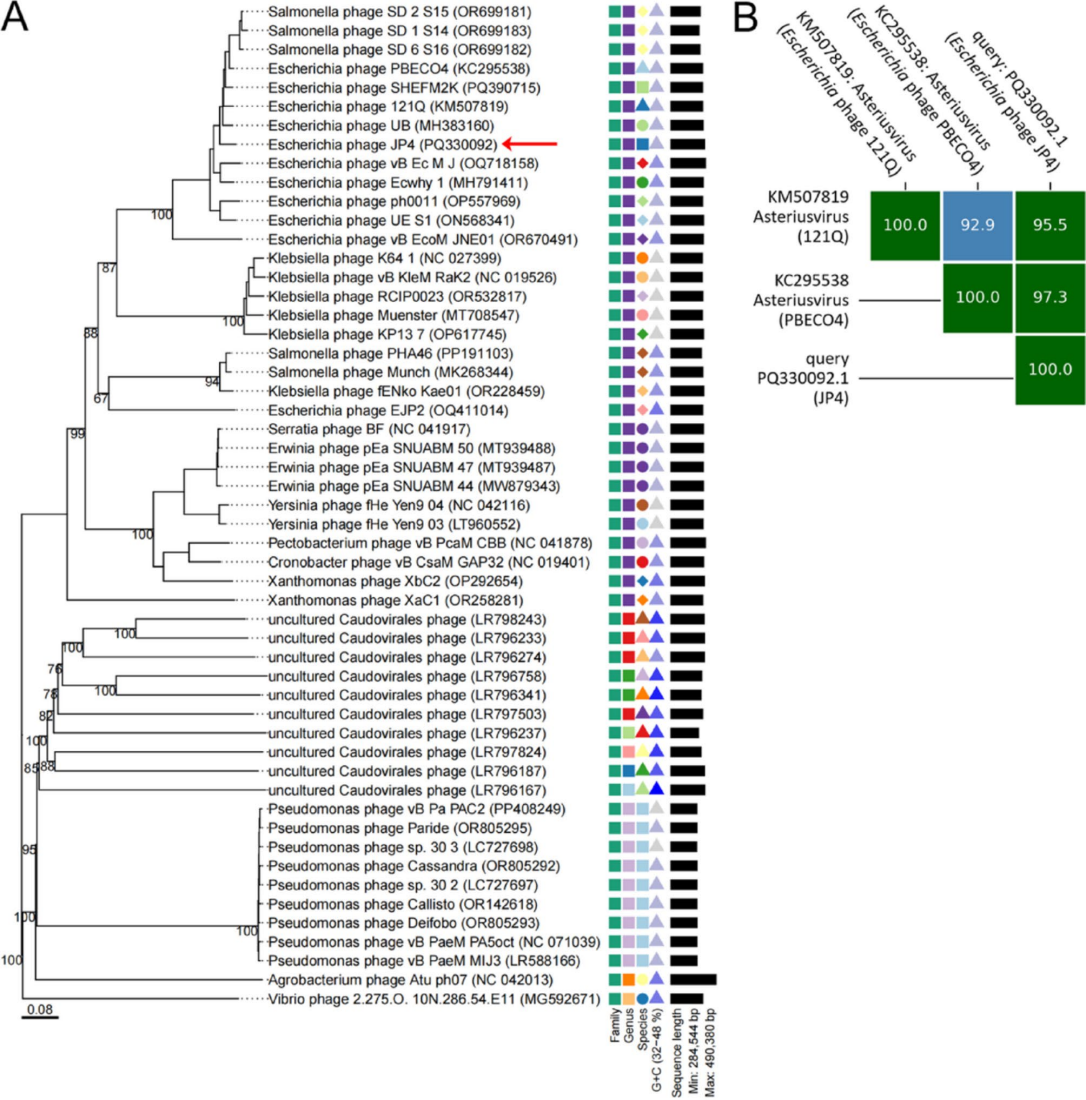
Phylogenetic and taxonomic analysis of phage JP4. **A** Phylogenetic analysis. The red arrow indicates phage JP4. Scale bar: relative genetic distance (0.08). **B** Taxonomic analysis using taxMyPhage (https://ptax.ku.dk/). The numbers in the color blocks represent the ANI percentage

Initial BlastN screening showed no significant nucleotide-level matches to existing sequences, underscoring the phage’s genomic novelty. Comparison with the NCBI Viral RefSeq database (accessed March 11, 2025) and the PhageScope database (https://phagescope.deepomics.org/) revealed potential evolutionary relationships between phage JP4 and multiple related phages. Comparative alignment of 20 genetically closest relatives identified *Escherichia* phagese PBECO4/121Q/UB/SHEFM2K and Salmonella phages SD-1_S14/SD-2_S15/SD-6_S16 as the nearest counterparts of phage JP4 (Fig. 5). Despite these relationships, the genome of JP4 displayed notable expansion, exceeding *Salmonella* phage SD-1_S14 by ~ 60 kb. Genomic architecture comparisons revealed conserved functional modules interspersed with structural variations including inversions, translocations, and deletions (Fig. 5). To better visualize the genomic sequence variations among these phages, a BRIG alignment was performed (Fig. 6). The results revealed that while these phages share notable similarities, they also exhibit distinct differences (represented as blank regions), which may reflect evolutionary recombination and selective pressures. Whole-genome phylogenetic reconstruction positioned JP4 closest to *Escherichia* phage UB/121Q/SHEFM2K/PBECO4 and *Salmonella* phages SD-1_S14/SD-2_S15/SD-6_S16, forming a distinct cluster (Fig. 7A). Notably, the phylogenetic topology demonstrated host-independent grouping patterns, with phages infecting different bacterial species clustering together on separate branches. This branching pattern suggests either horizontal genetic exchange events or conserved evolutionary trajectories across phage lineages. Taxonomic analysis using taxMyPhage revealed that phage JP4 shares average nucleotide identity (ANI) values exceeding 95% with members of the Asteriusvirus genus, including *Escherichia* phages 121Q and PBECO4 (Fig. 7B), satisfying the International Committee on Taxonomy of Viruses (ICTV) criteria for genus-level classification. The combined genomic, phylogenetic, and taxonomic evidence supports the classification JP4 as a novel species within Asteriusvirus while highlighting the complex evolutionary relationships between phages infecting enteric bacterial hosts.

### JP4 has strong in vitro antibacterial activity and biofilm clearance ability

**Fig. 8:**
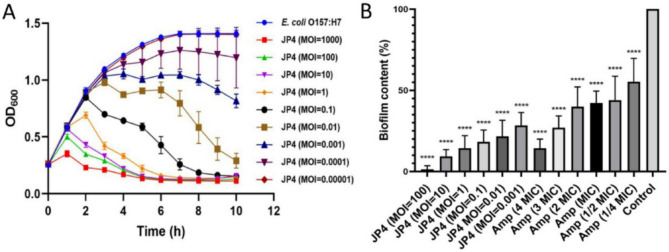
In vitro antibacterial activity and biofilm clearance ability of JP4. **A** In vitro antibacterial activity of JP4. **B** Biofilm clearance effect of JP4. **** represents *p* < 0.0001. Amp: Ampicillin

Without phage infections, the host bacteria exhibited rapid growth and entered the stationary phase within 7 h. Upon addition of phage JP4 at a MOI of 0.0001 or higher, the growth of the host bacteria was inhibited to varying extents. Phage JP4 revealed a significant bactericidal effect when the MOI reached or exceeded 0.1, and effectively suppressed the host bacterial population within 10 h (Fig. 8A).

Biofilm eradication assays showed that both phage JP4 with different MOIs and ampicillin with various MICs can partially or completely remove *E. coli* biofilms that had been established for over 48 h (Fig. 8B). All tested MOIs of JP4 showed significant biofilm clearance activity against *E. coli*. When the MOI of JP4 reached 10, more than 90% of the biofilm was removed (Fig. 8B). At a MOI of 0.001, JP4 exhibited biofilm clearance efficacy comparable to that of 3 MIC ampicillin, with both treatments removing over 70% of the biofilm (Fig. 8B).

## Discussion

The emergence of antibiotic-resistant Enterohemorrhagic *E. coli* (EHEC) poses significant public health risks, as these pathogens produce Shiga-like toxins causing severe gastrointestinal complications and potentially fatal hemolytic uremic syndrome [49]. The transfer of antimicrobial resistance genes through food chains has rendered conventional antibiotics increasingly ineffective against EHEC infections [50], necessitating alternative therapeutic strategies. Phages show particular promise due to their targeted bactericidal activity, with growing applications in clinical and food safety contexts [51]. Despite inherent challenges including limited host specificity and bacterial resistance development [52–54], phage therapy remains viable through continuous discovery of novel lytic phages. This study isolated a novel jumbo phage infecting *E. coli* O157:H7 from domestic sewage, which revealed a short latent period, strong in vitro bactericidal activity, and an effective ability to disrupt bacterial biofilms, suggesting its application potential in combating drug-resistant EHEC.

The environmental stability profile of phage JP4 enhances its practical utility across multiple application scenarios. Demonstrating good thermal resilience compared to other *E. coli*-targeting phages [26, 55], JP4 maintained infectivity at temperatures ≤ 60 °C – a critical advantage for storage and distribution logistics. Its broad pH tolerance (4–10) enables survival in diverse physiological environments, from gastric acidity to intestinal alkalinity, suggesting therapeutic viability through oral or systemic administration. While UV exposure caused progressive inactivation, JP4 retained 50% activity after 30-minute irradiation, indicating potential for combined UV-phage disinfection protocols in healthcare settings. This moderate sensitivity balances environmental persistence with controllable deactivation when required. The chloroform resistance confirms its structurally stable capsid lacking lipid envelopes, facilitating efficient concentration through chloroform-based purification methods – a crucial feature for industrial-scale production. These combined stability characteristics position JP4 as a robust candidate for both clinical applications and food safety interventions.

JP4 showed strict bacterial strain specificity and was unable to lyse Gram-negative bacteria such as *Shigella* and *Salmonella typhi*. Among 68 clinical isolates of *E. coli* (Table S1), JP4 can lyse three strains. This may be attributed to the highly specific receptor molecule of phage JP4. Further research is required to identify the receptor of phage JP4. The number of tested *E. coli* strains was not enough, and more clinical *E. coli* isolates should be analyzed to comprehensively determine the host range of JP4. New structural protein-coding genes can be identified via phage structural protein analysis [56]. Four novel structural protein-coding genes of phage JP4 were identified through SDS-PAGE and HPLC-MS. These genes lack predicted functional annotations and conserved domains; however, their presence in the virion suggests they encode structural components. While their precise functional roles—such as involvement in tail fiber formation, baseplate assembly, or DNA injection apparatus—remain to be elucidated through a series of genetic and biochemical experiments, this finding provides additional insights into the functional characterization of the numerous genes with unknown functions in phage JP4 genome.

The 370,741 bp genome establishes JP4 as a jumbo phage (≥ 200 kb) [57, 58], making it the largest known virus targeting *E. coli* O157:H7. While previously reported jumbo phages for this host, like sludge-derived vB_EcoM_EC001 (240,200 bp) [59], demonstrate size diversity, JP4 represents a genomic extreme. Analysis of NCBI Virus (https://www.ncbi.nlm.nih.gov/labs/virus/vssi/#/) data (accessed April 23, 2025) revealed that among >2,000 sequenced *Escherichia* phages, fewer than 30 exceed 200 kb (Fig. S2A). Globally, jumbo phages remain rare with ~ 600 sequenced ones, 80% spanning 200–300 kb (Fig. S2B). Host-based distribution analysis shows *Erwinia* phages dominate jumbo phage diversity (Fig. S2C), while *Escherichia*-specific jumbo phages rank seventh in prevalence. This contextualizes JP4’s significance as both a host-specific record-holder and a contributor to understanding jumbo phage evolution, positioning it as a valuable resource for both therapeutic development and viral genomics research.

To accommodate larger genomes, the head size of jumbo phages is typically larger than that of common phages. For example, the head diameter of another phage, vB_EcoS-UDF157lw, which can lyse *E. coli* O157:H7 and has a genome size of 46.6 kb, is approximately 55 nm [60], whereas the head diameter of phage JP4 reaches approximately 100 nm. This also suggested that jumbo phages may possess more complex structures and functions. Due to their large genomes and virion sizes, the isolation, identification, and in-depth study of jumbo phages are relatively challenging [61]. With advancements in high-throughput sequencing technology, metagenomics, and techniques for manipulating large DNA fragments, understanding of phage biological characteristics and their genomes has gradually deepened in recent years, leading to significant progress in jumbo phage research [19, 61]. Jumbo phages of varying sizes and from diverse sources have been continuously isolated and characterized [18]. Systematic and in-depth analysis of the rich genomes of jumbo phages has expanded and refined the functional gene library and evolutionary classification of phages [62].

## Conclusions

This study characterizes JP4, a novel jumbo phage targeting *E. coli* O157:H7, distinguished by its unusually large genome. Phage JP4 exhibited rapid replication kinetics and achieved complete bacterial eradication in vitro while disrupting mature biofilms, which are critical advantages for combating persistent infections. Its robust environmental stability and absence of virulence/antibiotic resistance genes position it as a safe therapeutic candidate. Genomic analysis revealed evolutionary kinship with *Escherichia* phage UB while identifying four previously uncharacterized structural genes (*JP4_CDS0703/0087/0010/0670*), resolving prior functional annotations. Current findings establish foundational therapeutic potential of phage JP4. By characterizing a novel jumbo phage within the Asteriusvirus genus, this work enhances our understanding of the diversity of jumbo phages, revealing the therapeutic promise of jumbo phage JP4 against challenging pathogens.

## Supplementary Information

Supplementary Material 1

Supplementary Material 2

Supplementary Material 3

Supplementary Material 4

## Data Availability

The complete genome sequence and annotations of phage JP4 have been deposited in GenBank under the accession number PQ330092.

## Abbreviations

WHO: World health organization
EPEC: Enteropathogenic E. coli
ETEC: Enterotoxigenic E. coli
EHEC: Enterohemorrhagic E. coli
PFUs: Plaque-forming units
EOP: Efficiency of plating
CFU: Colony-forming unit
LB: Luria–Bertani
OD: Optical density
TM: Tris-magnesium
TEM: Transmission electron microscopy
MOI: Multiplicities of infection
EDTA: Ethylenediaminetetraacetic acid
SDS: sodium dodecyl sulfate
NCBI: National center for biotechnology information
PAGE: Polyacrylamide gel electrophoresis
MIC: Minimum inhibitory concentration
ANOVA: Analysis of variance
CDS: Coding sequence
MW: Molecular weight
HPLC-MS: High-performance liquid chromatography-mass spectrometry
ANI: Average nucleotide identity
ICTV: the International committee on taxonomy of viruses
