# Supplementary material for: Characterization of a novel Jumbo phage JP4 with potential to control pathogenic Escherichia coli
Source: Virol J. 2025 Nov 25;22:386. doi: 10.1186/s12985-025-03001-4 (PMC12648861; doi:10.1186/s12985-025-03001-4)
Supplement: Supplementary file 1 — Supplementary Material 1 [file 12985_2025_3001_MOESM1_ESM.docx]

Table S1. Lysis of different bacterial strains by JP4

| Number | Strain name | Source* | JP4 |
| --- | --- | --- | --- |
| 1 | *Escherichia coli* 20140710142 | Department of laboratory medicine, Southwest Hospital (Clinical isolates) | - |
| 2 | *Escherichia coli*  20150412125 |  | - |
| 3 | *Escherichia coli*  THR01 |  | - |
| 4 | *Escherichia coli*  P9914 |  | - |
| 5 | *Escherichia coli*  THR05 |  | - |
| 6 | *Escherichia coli*  20140911021 |  | - |
| 7 | *Escherichia coli*  20141011083 |  | - |
| 8 | *Escherichia coli*  20151105076 |  | - |
| 9 | *Escherichia coli*  20141104361 |  | - |
| 10 | *Escherichia coli*  T9625 |  | - |
| 11 | *Escherichia coli*  P12656 |  | - |

| **12** | ***Escherichia coli***  **20151005076** | Department of laboratory medicine, Southwest Hospital  (Clinical isolates) | **+** |
| --- | --- | --- | --- |
| 13 | *Escherichia coli*  20140719026 |  | - |
| 14 | *Escherichia coli*  20140710140 |  | - |
| 15 | *Escherichia coli*  THR03 |  | - |
| 16 | *Escherichia coli*  20140923143 |  | - |
| 17 | *Escherichia coli*  20151115024 |  | - |
| 18 | *Escherichia coli*  20141102113 |  | - |
| 19 | *Escherichia coli*  20151038270 |  | - |
| 20 | *Escherichia coli*  20150812217 |  | - |
| 21 | *Escherichia coli*  20140520184 |  | - |
| 22 | *Escherichia coli*  20151211052 |  | - |
| 23 | *Escherichia coli*  20160407199 |  | - |
| 24 | *Escherichia coli*  THR02 |  | - |
| 25 | *Escherichia coli*  T11091 |  | - |
| 26 | *Escherichia coli*  IJ5880 | Department of laboratory medicine, Southwest Hospital  (Clinical isolates) | - |
| 27 | *Escherichia coli*  P1869 |  | - |
| 28 | *Escherichia coli*  20160209034 |  | - |
| 29 | *Escherichia coli*  P5618 |  | - |
| 30 | *Escherichia coli*  T5282 |  | - |
| 31 | *Escherichia coli*  20140902202 |  | - |
| 32 | *Escherichia coli*  20140508045 |  | - |
| 33 | *Escherichia coli*  20151101073 |  | - |
| 34 | *Escherichia coli*  IJ4057 |  | - |
| 35 | *Escherichia coli*  20160307144 |  | - |
| 36 | *Escherichia coli*  T5676 |  | - |
| 37 | *Escherichia coli*  20160413108 |  | - |
| 38 | *Escherichia coli*  20151029122 |  | - |
| 39 | *Escherichia coli*  P3463 |  | - |

| 40 | *Escherichia coli*  20160410092 | Department of laboratory medicine, Southwest Hospital  (Clinical isolates) | - |
| --- | --- | --- | --- |
| 41 | *Escherichia coli*  20151219055 |  | - |
| 42 | *Escherichia coli*  20160219293 |  | - |
| 43 | *Escherichia coli*  20151109179 |  | - |
| 44 | *Escherichia coli*  20160308150 |  | - |
| 45 | *Escherichia coli*  20151023258 |  | - |
| 46 | *Escherichia coli*  P16253 |  | - |
| 47 | *Escherichia coli*  20160220158 |  | - |
| 48 | *Escherichia coli*  20151226212 |  | - |
| 49 | *Escherichia coli*  2406291007  A5-3-D-32 | Department of laboratory medicine, Daping Hospital  (Clinical isolates) | - |
| 50 | *Escherichia coli*  2401034001  A3-5-B-79 |  | - |
| 51 | *Escherichia coli*  2401241074  A4-1-B-57 |  | - |

| 52 | *Escherichia coli*  2406081025  A5-2-D-46 | Department of laboratory medicine, Daping Hospital  (Clinical isolates) | - |
| --- | --- | --- | --- |
| 53 | *Escherichia coli*  2406031101  A5-2-C-23 |  | - |
| 54 | *Escherichia coli*  24-01241097  A4-1-B-64 |  | - |
| 55 | *Escherichia coli*  2401091105  A3-5-C-80 |  | - |
| 56 | *Escherichia coli*  2407041024  A5-4-A-2 |  | - |
| 57 | *Escherichia coli*  2403211089  A4-3-D-42 |  | - |
| 58 | *Escherichia coli*  2405251002  A5-1-E-81 |  | - |
| 59 | *Escherichia coli*  2401171086  A3-5-E-87 |  | - |
| 60 | *Escherichia coli*  240810045  A5-5-D-69 |  | - |

| 61 | *Escherichia coli*  2401021050  A4-5-B-80 | Department of laboratory medicine, Daping Hospital  (Clinical isolates) | - |
| --- | --- | --- | --- |
| 62 | *Escherichia coli*  2405311026  A5-2-B-73 |  | - |
| 63 | *Escherichia coli*  2401171027  A3-5-E-88 |  | - |
| 64 | *Escherichia coli*  2401311072  A4-1-D-86 |  | - |
| **65** | ***Escherichia coli***  **ER25-05-16A** |  | **+** |
| 66 | Escherichia coli  BR25-05-78A |  | - |
| 67 | Escherichia coli  DR25-04-19A |  | - |
| **68** | **Escherichia coli**  **ER25-06-13B** |  | **+** |
| 69 | *Escherichia coli* O157:H7 | Department of Microbiology, College of Basic Medical Sciences, Army Medical University  (Laboratory preservation) | + |
| 70 | *Shigella dysenteriae*  G1.126 |  | - |
| 71 | *Salmonella typhi* Ty2 |  | - |
| 72 | *Staphylococcus aureus* Newman |  | - |

*All located in Chongqing, China; +: lysis; -: no lysis.
