# Supplementary material for: Characterization of a novel Jumbo phage JP4 with potential to control pathogenic Escherichia coli
Source: Virol J. 2025 Nov 25;22:386. doi: 10.1186/s12985-025-03001-4 (PMC12648861; doi:10.1186/s12985-025-03001-4)
Supplement: Supplementary file 3 — Supplementary Material 3 [file 12985_2025_3001_MOESM3_ESM.pdf]

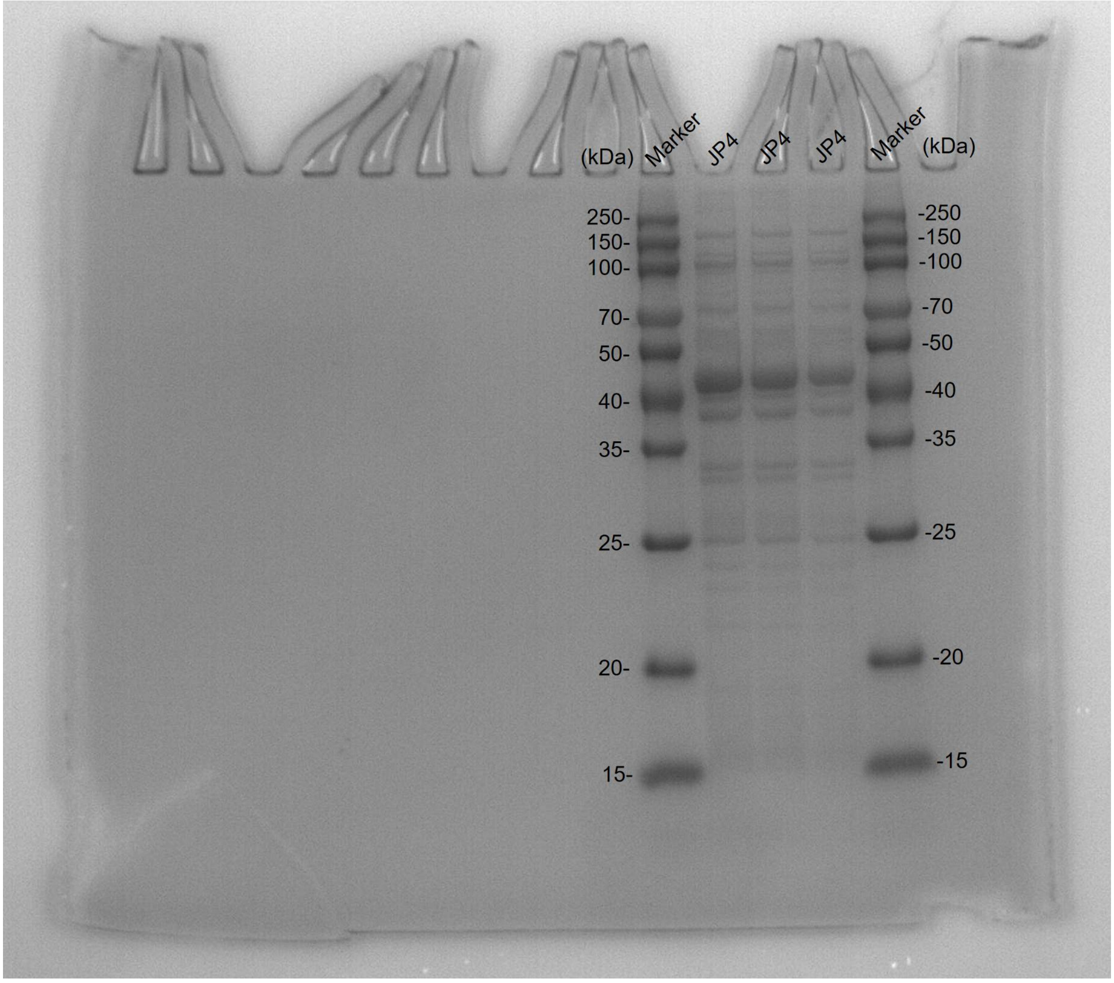

Fig. S1. The original uncropped image of SDS-PAGE analysis for identification of JP4 structural proteins.
