# Supplementary material for: Characterization of a novel Jumbo phage JP4 with potential to control pathogenic Escherichia coli
Source: Virol J. 2025 Nov 25;22:386. doi: 10.1186/s12985-025-03001-4 (PMC12648861; doi:10.1186/s12985-025-03001-4)
Supplement: Supplementary file 4 — Supplementary Material 4 [file 12985_2025_3001_MOESM4_ESM.pdf]

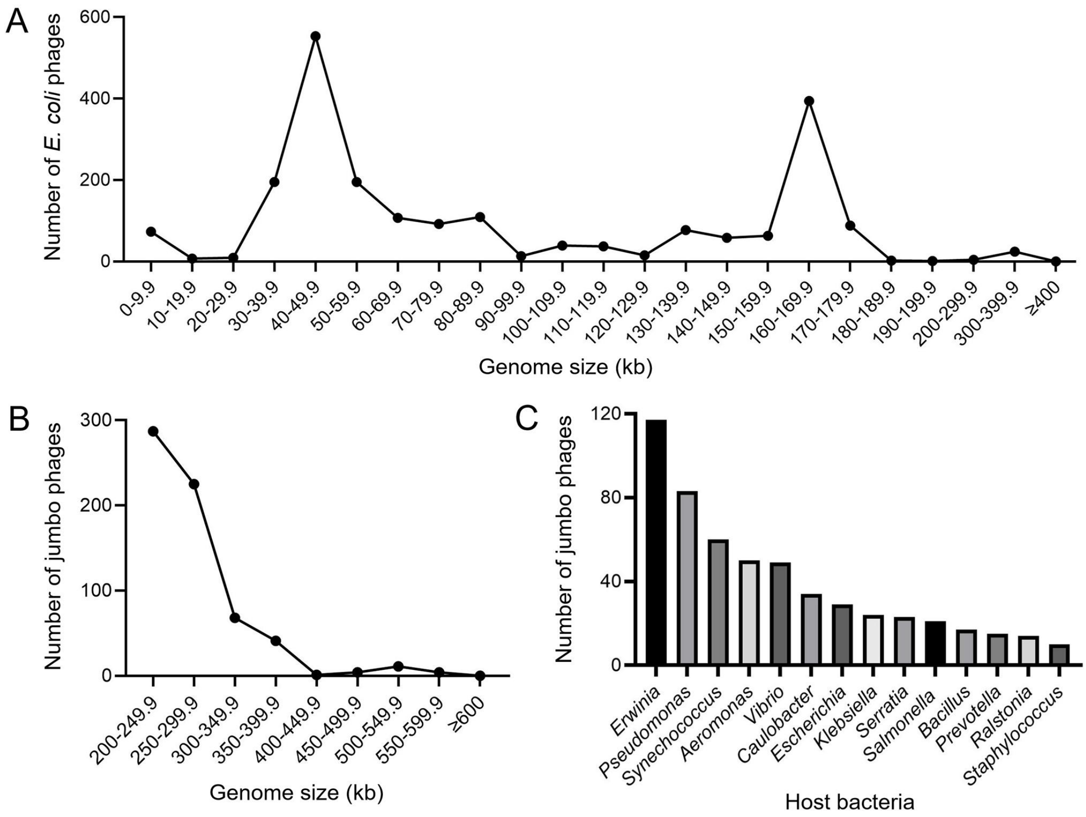

Fig. S2. Statistics of phage quantities. **A** Quantity distribution of *E. coli* phages according to genome size. **B** Quantity distribution of jumbo phages according to genome size. **C** Classification statistics of jumbo phages according to host bacteria. Bacterial genera with more than 10 jumbo phages are shown.
